# Circulating Lipid Profiles Indicate Incomplete Metabolic Recovery After Weight Loss, Suggesting the Need for Additional Interventions in Severe Obesity

**DOI:** 10.3390/biom15081112

**Published:** 2025-08-01

**Authors:** Alina-Iuliana Onoiu, Vicente Cambra-Cortés, Andrea Jiménez-Franco, Anna Hernández-Aguilera, David Parada, Francesc Riu, Antonio Zorzano, Jordi Camps, Jorge Joven

**Affiliations:** 1 Unitat de Recerca Biomèdica, Hospital Universitari de Sant Joan, Institut d’Investigació Sanitària Pere Virgili, Universitat Rovira i Virgili, Av. Dr. Josep Laporte 2, 43204 Reus, Spain; alinaiuliana.onoiu@urv.cat (A.-I.O.); vicente.cambra@urv.cat (V.C.-C.); andrea.jimenez@urv.cat (A.J.-F.); 2 Department of Pathology, Hospital Universitari de Sant Joan, Institut d’Investigació Sanitària Pere Virgili, Universitat Rovira i Virgili, Av. Dr. Josep Laporte 2, 43204 Reus, Spain; anna.hernandez@salutsantjoan.cat (A.H.-A.); david.parada@salutsantjoan.cat (D.P.); francesc.riu@salutsantjoan.cat (F.R.); 3 Institute for Research in Biomedicine, Department of Biochemistry and Molecular Medicine, Universitat de Barcelona, C. Baldiri Reixac 10, 08028 Barcelona, Spain; antonio.zorzano@irbbarcelona.org

**Keywords:** biomarkers, fatty acid metabolism, lipidomics, obesity, oxylipins, metabolic surgery, precision medicine

## Abstract

The effects of long-term adjustments in body weight on the lipid balance in patients with severe obesity are not well understood. This study aimed to evaluate a non-invasive lipidomic approach to identifying biomarkers that could help predict which patients may require additional therapies before and after weight loss. Using mass spectrometry, 275 lipid species were analysed in non-obese controls, patients with severe obesity, and patients one year after bariatric surgery. The results showed that severe obesity disrupts lipid pathways, contributing to lipotoxicity, inflammation, mitochondrial stress, and abnormal lipid metabolism. Although weight loss improved these disturbances, surgery did not fully normalise the lipid profiles of all patients. Outcomes varied depending on their baseline liver health and genetic differences. Persistent alterations in cholesterol handling, membrane composition, and mitochondrial function were observed in partial responders. Elevated levels of sterol lipids, glycerophospholipids, and sphingolipids emerged as markers of complete metabolic recovery, identifying candidates for targeted post-surgical interventions. These findings support the use of lipidomics to personalise obesity treatment and follow-up.

## 1. Introduction

Obesity and its related abnormalities are the most serious threats to public health worldwide [1,2]. Obesity is a chronic and systemic disease characterised by excessive fat accumulation, which disrupts the normal functioning of tissues and organs [3]. The consequences of obesity are complex, and many aspects remain poorly understood. To effectively mitigate its metabolic impact, proactive prevention strategies are essential. When these strategies are insufficient, targeted medications and aggressive weight loss interventions become necessary [4,5,6,7]. A specific group of patients suffering from severe obesity, defined as having a body mass index (BMI) of 40 kg/m^2^ or greater, requires special attention due to their exceptionally high risk of life-threatening complications [8,9].

These patients provide a valuable model for studying how the circulating lipidome reflects metabolic abnormalities caused by changes in fat accumulation and weight loss [10,11,12,13]. Many conventional weight-loss strategies lose effectiveness over time. In patients with severe obesity, weight loss achieved through lifestyle changes is often not sustained. Furthermore, there is limited information on the role of newly available medications in the management of this disease [14]. In contrast, laparoscopic sleeve gastrectomy (LSG) and other more invasive surgical procedures can lead to rapid, significant, and sustained weight loss, with effects observable at the cellular level [15,16,17]. These surgeries lead to systemic metabolic changes [18,19] and highlight the crucial roles of the liver and adipose tissue in regulating the complex lipid metabolism processes associated with obesity [20,21,22,23,24,25]. The effect of weight loss on circulating lipid levels has not been thoroughly investigated, primarily due to the significant challenges related to studying the diversity of human lipids. For instance, the most widely accepted classification system identifies over 50,000 unique lipid structures, many of which exist in tiny quantities. As a result, investigators will need to evaluate the vast amount of information generated from lipidomics critically. A practical research approach should combine high-throughput lipidomics methods with computational data processing tools and clinico-biological information [18,19,26,27]. In this study, we capitalise on the weight loss observed after LSG in patients with severe obesity to investigate their disrupted lipid metabolism and the impact of weight loss on improving metabolic health. Our findings may contribute to personalised management strategies for obesity-related conditions.

## 2. Materials and Methods

### 2.1. Ethics and Participants

This pilot study received approval from our institution’s ethics committee (EPIMET PI21/00510_083, PL4NASH112/2021, and EOM 244/2024) in accordance with the procedures outlined in the Declaration of Helsinki. All participants provided written informed consent.

We recruited 50 healthy, non-obese individuals from a population study conducted by the Institut d’Investigació Sanitària Pere Virgili [28]. This group, referred to as Cohort 1, followed standardised biobanking strategies and adhered to protocol adjustments for sample collection and maintenance. To ensure reliable results, blood samples were collected from all participants between 8:00 and 9:00 a.m., after requiring them to fast for at least 10 h and to refrain from physical activity for the last 30 min.

We enrolled 50 patients with severe obesity scheduled to undergo LSG who had maintained a stable weight for at least one month before surgery (Cohort 2). Blood samples from this cohort were collected during the week before surgery. To study the effects post-surgery, additional blood samples were collected during a scheduled visit exactly one year after the procedure (Cohort 3). These patients were recruited from a prospective longitudinal study registered at ClinicalTrials.org (document number: NCT05554224). All eligible patients met the inclusion criteria for LSG, which were as follows: patients over 18 years old, patients who were not pregnant or breastfeeding, patients who had undergone a psychiatric evaluation, and patients with a BMI of 35 kg/m^2^ or greater. The exclusion criteria included patients with clinical or analytical evidence of severe illness, chronic or acute inflammation, cancer, or infectious diseases. Our previous findings informed our approach to determining the sample size and matching criteria using the algorithm from the MatchIt R package [4,27,29]. All participants in the study shared a similar ethnic background and were recruited in line with the typical skewed sex distribution observed in our patient population, which consisted of 60% women and 40% men. To evaluate the effectiveness of LSG in reducing body weight and to investigate the associated metabolic differences, patients were arbitrarily classified as total responders if their post-surgical BMI fell below 35 kg/m^2^ and as partial responders if it remained above 35 kg/m^2^.

Venous blood was collected from all participants into EDTA-containing tubes for the isolation of plasma, which was used in lipidomic analyses, and the buffy coat, which was used for genotyping. Additional blood was collected into serum-separating tubes without anticoagulant for routine biochemical assessments. The samples were processed within two hours and stored at −80 °C until analysis, except for routine biochemical analyses, which were performed immediately using standard tests on a Roche Modular Analytics system (Roche Diagnostics, Basel, Switzerland). All clinical and surgical interventions were designed and implemented according to the most recent evidence-based guidelines for obesity treatment [30].

### 2.2. Liver Biopsies and Genotyping

The variation in surgical responses necessitates risk stratification based on baseline liver damage and genotypes that are known to influence the disease. Wedge liver biopsies were collected during surgery. These biopsies were stained using haematoxylin and eosin, as well as Masson’s trichrome stain. They were then scored using whole-slide imaging and a validated histological method to evaluate steatosis, hepatocellular ballooning, lobular inflammation, and fibrosis [31,32]. We extracted DNA from buffy coats to analyse the allele frequencies of gene polymorphisms using TaqMan™ SNP Genotyping Assays in an OpenArray AutoLoader instrument coupled to a QuantStudio 12K qPCR system (Thermo Fisher, Barcelona, Spain). Specifically, we assessed four SNPs of the fat mass and obesity-associated (*FTO*) gene (rs9939609, rs9930506, rs8050136, and rs17817449), as well as one SNP each from patatin-like phospholipase domain-containing protein 3 (*PNPLA3*, rs738409) and tribbles pseudokinase 1-associated lnc RNA (*TRIB1AL*, rs6982502). The *FTO* gene encodes an N6-methyladenosine demethylase RNA that plays a role in energy balance, appetite regulation, and adipogenesis. Genetic variants in *FTO* have been consistently associated with BMI and an increased risk of obesity [33]. The *PNPLA3* gene encodes a lipid droplet-associated protein involved in triglyceride hydrolysis in liver and adipose tissue. The variant I148M has been proposed to affect the release of lipids from lipid droplets causing lipid accumulation in liver [33]. *TRIB1AL* is a long non-coding RNA located near the tribbles pseudokinase 1 locus on chromosome 8q24.13. *TRIB1AL* has been associated with plasma triglyceride levels. Emerging evidence suggests it may regulate lipid metabolism, possibly impacting liver fat accumulation and cardiovascular risk [34].

### 2.3. Lipidomics Analyses

A total of 275 lipid species were identified in our analysis, including 62 fatty acyls, 33 glycerolipids, 127 glycerophospholipids, 25 sphingolipids, and 28 sterol lipids. The complete list of lipid species is provided in Supplementary Table S1. Details regarding the extraction columns, the composition of mobile phases and solvents, and the various gradients can be found elsewhere [27,35]. Additional experimental data, reference spectra, and analytical procedures are available through the Metabolights database and repository under the study number MTBLS7758. We obtained lipid extracts using either a mixture of tert-butyl ether and methanol in a 1:2 (*v*/*v*) ratio containing 0.5% acetic acid or simply methanol. The process involves sample preparation and analyses using a chromatography system that includes an ultra-high-pressure liquid chromatograph (UHPLC) coupled with a quadrupole-time-of-flight mass spectrometer (QTOF), which was equipped with an electrospray ionisation (ESI) source. This system, provided by Agilent Technologies (Santa Clara, CA, USA), consisted of a binary pump (G4220A) and an autosampler (G4226A), which was maintained at 4 °C. To ensure measurement reproducibility, we injected lipid extracts from a pool of different samples twice daily and conducted quality control checks after every 20 analyses. Labelled internal standards were from Cayman Chemical (Ann Arbor, MI, USA), Cambridge Isotope Laboratories (Andover, MA, USA), or the SPLASH mixture from Avanti Polar Lipids (Alabaster, AL, USA). We employed a semi-targeted approach, utilising selected standards from each lipid category to create calibration curves for quantifying the corresponding lipid species. We used a representative standard mixture from the same lipid class to measure lipids with similar chemical structures. Labelled internal standards were implemented to adjust the responses of each detected lipid species. To address potential drawbacks, we followed detailed protocols for lipidomic analysis [35,36,37], ensuring that accurate masses and isotopic distributions aligned with the Metlin PCDL database (Scripps Research Institute, La Jolla, CA, USA) and Lipid MAPS^®^. Quantification was performed using the Mass Hunter Quantitative Analysis B.07.00 software from Agilent Technologies (Santa Clara, CA, USA).

### 2.4. Statistical Analyses

Statistical analyses were conducted using RStudio (R version 4.0.2) and available modules in MetaboAnalystR 5.0. We utilised the Readxl and dplyr packages for data management. We performed the Shapiro–Wilk test to assess the normality of each variable’s distribution. To maintain consistency, we employed non-parametric methods in our descriptive statistics. The Mann–Whitney U and Kruskal–Wallis tests were used for comparisons between two groups and multiple groups, respectively. We applied the Fisher Exact test to categorical variables. The Tableone package helped us summarise relevant data from our cohorts, presenting continuous variables as medians and interquartile ranges and categorical variables as counts and percentages. For graphical representations, we used the ggplot2, ggpubr, and pROC packages to create box plots, bar plots, correlation plots, and Receiver Operating Characteristic (ROC) curves. When necessary, we performed additional analyses, including hierarchically clustered heatmaps, Partial Least Squares Discriminant Analysis (PLS-DA), random forest analysis, and biomarker and enrichment analysis using MetaboAnalyst. A Benjamini–Hochberg false discovery rate (FDR) *p*-value < 0.05 was considered significant for the overall analyses, and a Bonferroni *p*-value < 0.05 was used for pairwise comparisons.

## 3. Results

### 3.1. LSG Is Effective in Inducing Weight Loss

We included a group of healthy, non-obese individuals as controls to compare the changes in the biochemical variables of patients with severe obesity and to evaluate the effects of surgical weight loss. The characteristics of the participants (Table 1) highlighted the connection between excess body fat and metabolic health. One year after LSG, patients were still classified as having obesity, even though they experienced a 27% reduction in BMI. A decrease in the number of prescribed medications further indicated significant improvements in conditions such as diabetes, hypertension, and dyslipidaemia. Moreover, laboratory markers related to glucose and lipid metabolism, along with indicators of liver damage, returned to levels comparable to those of the control group. In parallel, non-invasive imaging techniques conducted on a subset of participants revealed significant differences in fat distribution among the groups (Supplementary Figure S1).

### 3.2. The Circulating Lipidome Captures the Systemic Metabolic Stress in Severe Obesity

**Figure 1:**
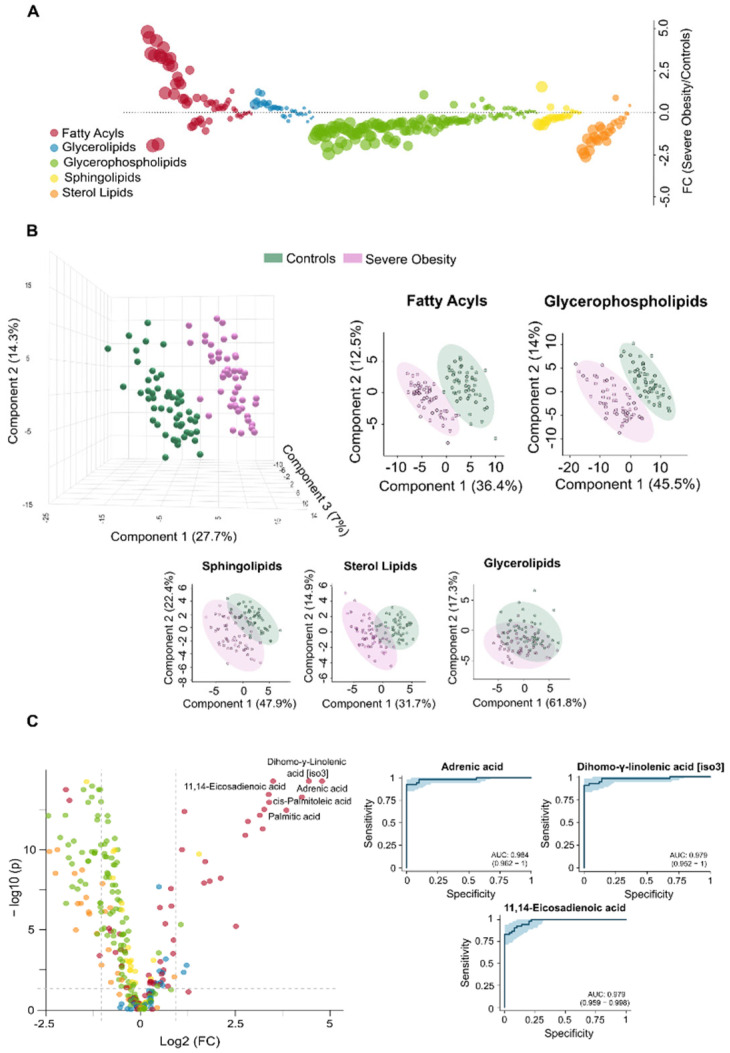
Severe obesity induces significant changes in the circulating lipidome. (**A**) The bubble plot illustrates the magnitude and direction of lipid alterations across different classes in individuals with severe obesity compared to controls. (**B**) The Partial Least Squares Discriminant Analysis shows a clear distinction between the control group and individuals with severe obesity across various lipid classes, with fatty acyls and glycerophospholipids demonstrating the strongest ability to differentiate between the groups. (**C**) The volcano plot displays the individual lipids showing the greatest differences between groups, and the Receiver Operating Characteristic curves for the three most significantly altered species, highlight their potential as diagnostic indicators of metabolic stress. AUC: Area under the curve; FC: Fold change.

**Figure 2:**
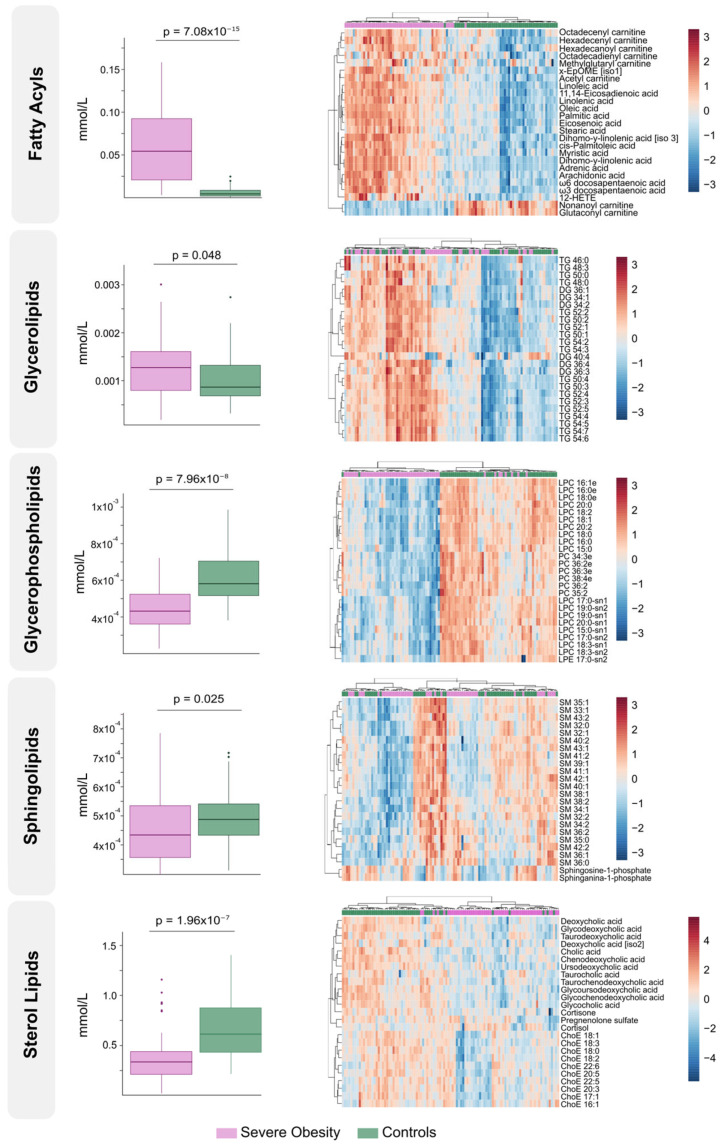
Class-specific lipid alterations and individual species expression patterns in severe obesity. The left side displays box plots comparing total lipid class concentrations between the severe obesity group and the control group. On the right, heatmaps illustrate the most differentially expressed lipid species within each lipid class, with hierarchical clustering showing distinct patterns of regulation. ChoE: Cholesterol ester; DG: Diglyceride; EpOME: Epoxide form of linoleic acid; HETE: Hydroxyeicosatetraenoic acid; LPC: Lysophosphatidylcholine; LPE: Lysophosphatidylethanolamine; PC: Phosphatidylcholine; SM: Sphingomyelin; TG: Triglyceride.

The differences found in circulating lipidome between non-obese individuals and patients with severe obesity reflect a complex dysregulation. Patients with severe obesity exhibited significantly higher levels of circulating fatty acyls and glycerolipids. In contrast, the concentrations of glycerophospholipids, sphingolipids, and sterol lipids were markedly lower (Figure 1A). PLS-DA demonstrated that plasma lipidomic signatures effectively distinguish individuals with severe obesity from non-obese controls (Figure 1B). Figure 2 presents box plots and heatmaps illustrating differences in the lipid subclass concentrations and highlighting the lipid species showing the most significant variation within each subclass. Notably, individuals with severe obesity showed a marked reduction in bile acids and cholesteryl esters within the sterol lipid subclass, suggesting disruptions in bile acid circulation. Differentially abundant lipids are ranked by statistical significance in Supplementary Table S2. Volcano plots and a hierarchical cluster analysis further confirm distinct lipidomic profiles at the species level (Figure 1C), supported by variable importance in projection (VIP) scores (Supplementary Figure S2A), which identify the most discriminative lipid species. Several of these, particularly those involving polyunsaturated fatty acids and eicosanoid metabolism, such as adrenic, 11,14-eicosadienoic, and linoleic acids, show promise as binary classifiers (Figure 1C). Overall, the pronounced remodelling of circulating lipid profiles in severe obesity underscores underlying oxidative, inflammatory, and mitochondrial stress, pointing to wide-ranging biological consequences.

### 3.3. Extensive Weight Loss Causes Dynamic Changes in the Circulating Lipidome

**Figure 3:**
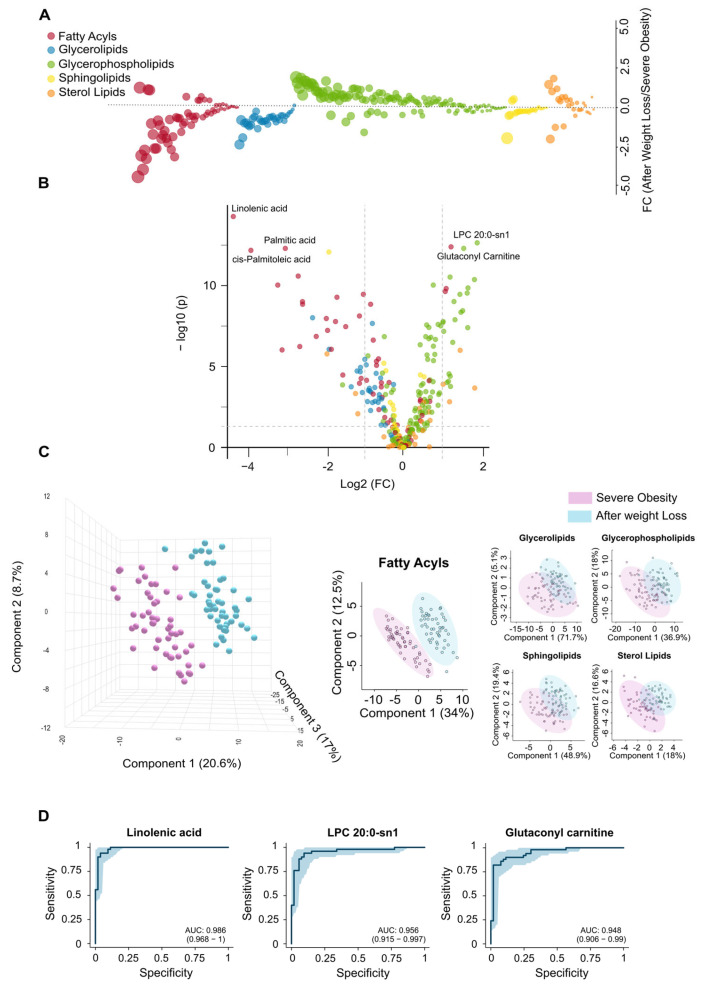
Surgery-induced weight loss partially restores changes in the circulating lipidome. (**A**) The bubble plot compares the alterations in lipid classes in individuals with severe obesity before and after weight loss, illustrating the extent of restoration in the circulating lipidome following bariatric surgery. (**B**) The volcano plot displays the individual lipids showing the greatest differences between groups. (**C**) Partial Least Squares Discriminant Analysis shows a clear separation between the groups, with fatty acyls providing the most significant differentiation. (**D**) Receiver Operating Characteristic curves demonstrate the potential of the most significantly altered lipid species as biomarkers following the bariatric intervention. AUC: Area under the curve; FC: Fold change; LPC: Lysophosphatidylcholine.

Complementing the clinical observations in patients who underwent LSG (Table 1, Supplementary Figure S1), the lipid changes observed after one year reflect a significant shift in lipidomics toward a healthier metabolic state. Fatty acyls and glycerolipids, which were elevated in obese patients compared to controls (Figure 1A), tended to normalise after weight loss. In contrast, glycerophospholipids and sterol lipids, initially reduced, showed a tendency to increase (Figure 3A). Supplementary Figure S3 displays box plots and heatmaps that depict variations in lipid subclass concentrations and emphasise the lipid species with the most pronounced changes within each subclass. The volcano plot in Figure 3B highlights the five most significantly altered lipid species after LSG based on the fold change and *p*-values. These include reduced levels of the fatty acids linolenic, palmitic, and palmitoleic acids, alongside increased levels of the glycerophospholipid lysophosphatidylcholine and the carnitine ester glutaconyl carnitine. Our results from the PLS-DA indicate that lipid profiles can effectively distinguish patients following weight loss, with differences in fatty acids playing a primary role in this differentiation (Figure 3C).

The results presented in Supplementary Table S2 further support and expand these findings. Following surgery, we observed a significant reduction in polyunsaturated fatty acids, which serve as precursors to eicosanoids, suggesting a decrease in chronic low-grade inflammation. Reductions in saturated and monounsaturated fatty acids, including palmitic, myristic, and oleic acids, point to diminished hepatic lipid synthesis. Weight loss also reversed the accumulation pattern of several acylcarnitines, indicating enhanced mitochondrial efficiency and reduced lipotoxicity. The restoration of lysophosphatidylcholines (LPCs) and lysophosphatidylethanolamines (LPEs) reflects improved phospholipid turnover, which may contribute to normalised lipoprotein metabolism. Moreover, decreased levels of sphingomyelin (SM) and complex triglycerides suggest a reduction in lipid storage and lipotoxic intermediates, supporting systemic metabolic recovery, improved insulin sensitivity, and a lower cardiometabolic risk profile. Finally, the analysis of VIP scores (Supplementary Figure S2B), the actual values presented in Supplementary Table S2, and the ROC curves (Figure 3D) have identified linolenic acid, LPC 20:0-sn1, and glutaconyl carnitine as potential markers for assessing the metabolic benefits of significant weight loss.

### 3.4. Lipidomic Signatures Indicate Persistent Dysregulation After Weight Loss

**Figure 4:**
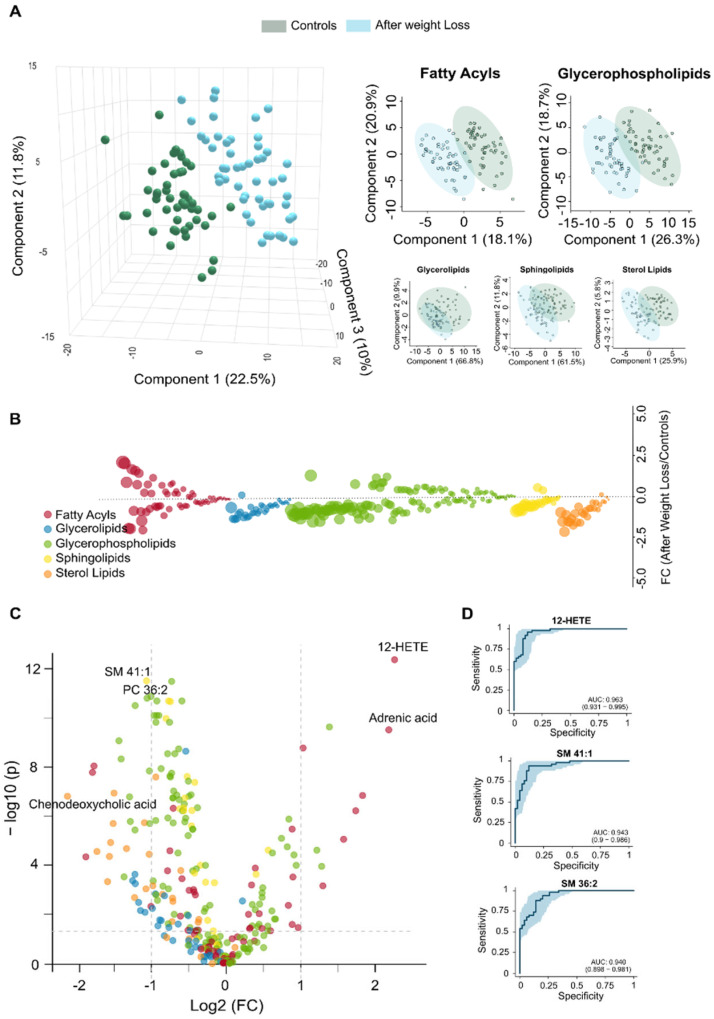
Comparisons in the circulating lipidome between post-surgical patients and control group confirm metabolic recovery. (**A**) The two groups show a modest separation in the Partial Least Squares Discriminant Analysis, with no lipid family exhibiting complete separation. (**B**) The bubble plot displays the magnitude and direction of remaining lipid alterations across families. (**C**) The volcano plot illustrates the differentially abundant lipids species between the two groups. (**D**) Receiver Operating Characteristic analysis highlights the top three discriminating lipid biomarkers, revealing residual alterations in lipid homeostasis. AUC: Area under the curve; FC: Fold change; HETE: Hydroxyeicosatetraenoic acid; SM: Sphingomyelin.

Despite substantial improvements following weight loss, the circulating lipidome does not completely revert to the profile of the non-obese cohort (Figure 4). Our results support the idea that LSG leads to systemic metabolic recovery; however, PLS-DA indicates residual differences (Figure 4A). These differences may be partly explained by the finding that patients who underwent surgery remained classified as obese (Table 1). Our findings highlight qualitative changes suggesting that metabolic reprogramming is still in progress. The distribution of glycerolipids remained nearly identical between the two groups, while plasma bile acids levels were either decreased or returned to near-normal levels (Supplementary Figure S4). Additionally, there was considerable variability in other lipid classes (Supplementary Table S2, Supplementary Figure S4). The bubble and volcano plots show that the lipid species with the greatest changes following weight loss were downregulated, although some were significantly upregulated (Figure 4B,C). The differences observed in glycerophospholipids, including phosphatidylcholines (PCs), ether-linked PCs, and LPEs, indicate ongoing disruptions in phospholipid remodelling and biosynthesis. These disruptions, as well as those shown in Supplementary Table S2, suggest persistent alterations in membrane integrity and lipid-mediated signalling, particularly in specific species such as PC 36:2, PC 36:3e, PC 34:2, and LPE 22:4-sn2. We noted a decrease in sphingomyelins, such as SM 41:1, SM 41:2, and SM 39:1, indicating a delayed normalisation of sphingolipid pathways, which may be related to effects on membrane dynamics. Remarkably, adrenic acid and oxylipins levels, like 12-hydroxyeicosatetraenoic acid (12-HETE) and 9,12,13-trihydroxy-10-octadecenoic acid (9,12,13-TriHOME), were elevated compared to those in the controls, indicating adaptive immune remodelling in a context of residual systemic inflammation. The lower circulating concentration of octanoyl carnitine after weight loss suggests a reduced accumulation of intermediates in mitochondrial β-oxidation. The actual concentrations listed in Supplementary Table S2 and the calculated VIP scores shown in Figure S2C highlight potential candidates for assessing variability. According to the ROC curves, plasma levels of 12-HETE, SM 41:1, and SM 36:2 may serve as biomarkers for partial normalisation, as illustrated in Figure 4D. These comparisons suggest that specific metabolic pathways remain partially dysregulated after surgery. Alternatively, a previous metabolic history may limit the complete restoration of metabolism, requiring longer-term adaptation.

### 3.5. Residual Dysregulation Supports the Use of Lipidomic Biomarkers for Prediction of Outcomes and Long-Term Follow-Up

**Figure 5:**
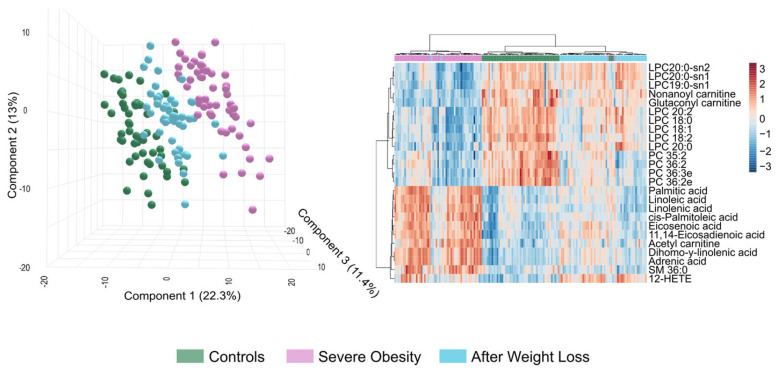
Post-bariatric surgery patients exhibit an intermediate lipidome profile between the control and severe obesity groups. The Partial Least Squares Discriminant Analysis on the left illustrates the trajectory of metabolic changes from the disease state to surgical intervention. The heatmap on the right displays the expression patterns of the most differentially regulated lipid species across all three groups, highlighting distinct clustering patterns. This analysis demonstrates both the metabolic disruption caused by severe obesity and the partial normalisation achieved through weight loss interventions. HETE: Hydroxyeicosatetraenoic acid; LPC: Lysophosphatidylcholine; PC: Phosphatidylcholine; SM: Sphingomyelin.

Despite achieving a healthier lipid profile, LSG does not entirely reverse the lipid changes associated with severe obesity in all patients. The lipidomic profiles of patients who have lost weight fall between those of individuals with severe obesity and non-obese controls. Our findings reveal different expression patterns in lipid species across all three groups, highlighting distinct clustering patterns (Figure 5). This analysis underscores the metabolic disruption caused by severe obesity and the partial normalisation achieved through weight loss interventions. However, the patient responses were not uniform. When comparing total and partial responders, we found that total responders had a significantly lower baseline body weight and experienced greater postoperative weight loss. They also exhibited a trend toward higher statin use (Supplementary Table S3). Notably, despite comparable conventional biochemical parameters, the two groups displayed distinct lipidomic profiles. These findings suggest that routine clinical measurements may fail to capture key alterations in underlying metabolic pathways. Therefore, the use of new biomarkers may be considered to inform the development of additional therapeutic interventions.

**Figure 6:**
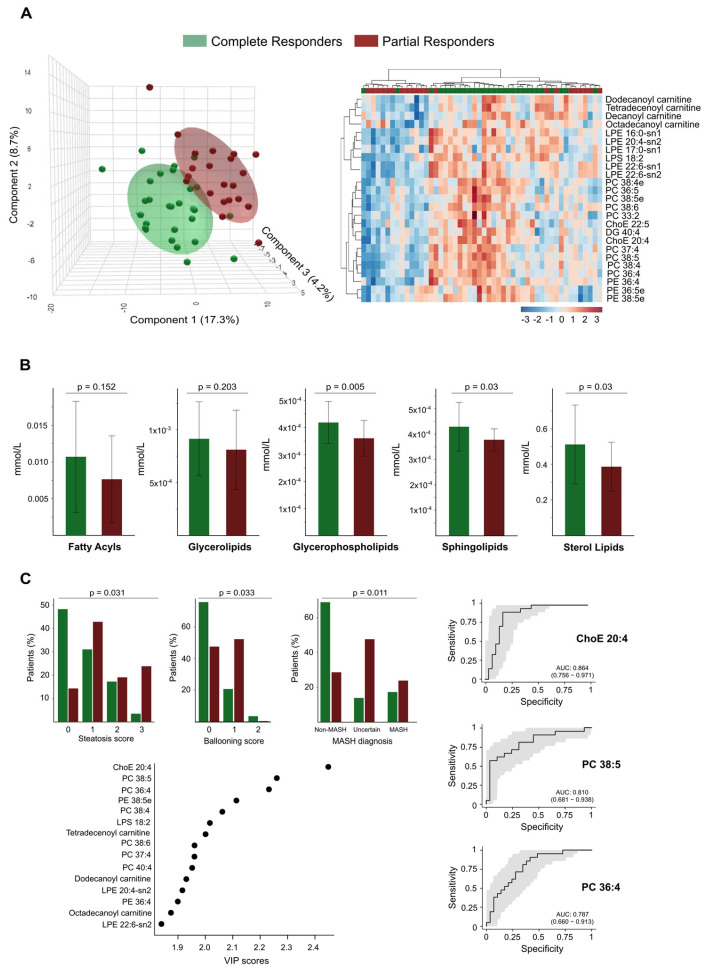
Lipidomic profiling differentiates complete from partial responders and correlates with histological and diagnostic features. (**A**) The Partial Least Squares Discriminant Analysis (on the left) and the heatmap (on the right) illustrate the distinct separation between complete responders and partial responders based on their plasma lipidomic profiles. The heatmap highlights the top discriminant lipid species. (**B**) Bar plots compare the abundances of lipid classes between complete and partial responders. Significant differences are observed in glycerophospholipids, sphingolipids, and sterol lipids. (**C**) The associations between lipidomic profiles and histological features, such as steatosis score, ballooning score, and metabolic dysfunction-associated steatohepatitis diagnosis, are also noted. A Variable Importance in the Projection (VIP) plot identifies the lipid species that contribute most to group separation. Receiver Operating Characteristic curves for key lipid species demonstrate strong diagnostic performance. AUC: Area under the curve; ChoE: Cholesterol ester; DG: Diglyceride; LPE: Lysophosphatidylethanolamine; PC: Phosphatidylcholine; PE: Phosphatidylethanolamine.

Through PLS-DA and hierarchical cluster analysis of the circulating lipidome profile, we were able to differentiate between the two groups at this follow-up stage (Figure 6A). We identified significant differences in the plasma concentrations of 68 lipid species between the groups, with higher concentrations found in total responders (Supplementary Table S4). Notably, we observed no differences in the concentrations of circulating fatty acids between the groups; we found the most significant differences in glycerophospholipids. The levels of the glycerolipid diacylglycerol (DG) 40:4 were also higher among total responders (Supplementary Table S4). Greater BMI reduction was also associated with milder baseline liver damage; steatosis, hepatocellular ballooning, and metabolic dysfunction-associated steatohepatitis diagnosis were less frequent among responders (Figure 6C). These findings align with the lower prevalence of risk-associated polymorphisms in total responders, specifically in genes linked to obesity (*FTO*), liver injury (*PNPLA3*), and cardiometabolic risk (*TRIB1AL*), as shown in Supplementary Figure S5. Further analysis using VIP scores identified several lipid species, such as the cholesteryl arachinodate (ChoE 20:4), PC 38:5, and PC 36:4, as effective discriminators between total and partial responders. ROC curve analysis confirmed their predictive potential, with AUC values of 0.86, 0.81, and 0.79, respectively (Figure 6C).

## 4. Discussion

Our study offers valuable insights into the dynamic changes in lipid homeostasis after one year of management and follow-up in patients with severe obesity. We emphasise that treatment should focus on achieving metabolic normalisation rather than solely on weight loss. Some patients may continue to experience dysregulation in specific metabolic pathways. Our findings suggest that lipidomic markers can help in the early identification of individuals who are not responding optimally.

Our observations align with the concept of lipid-induced chronic, low-grade inflammation and oxidative stress [38,39]. Specifically, we found a strong association between severe obesity and the plasma levels of cis-7,10,13,16-docosatetraenoic acid, commonly known as adrenic acid. Its catabolism, through both enzymatic and non-enzymatic reactions, produces lipid derivatives with distinct biological effects on inflammation, oxidative stress, and cell death [40]. We also observed significant increases in the levels of cis-palmitoleic acid, palmitic acid, and oleic acid. These results suggest the presence of lipotoxicity and changes in de novo lipogenesis [41]. In particular, palmitic acid, a saturated fatty acid, may contribute to insulin resistance and promote inflammatory pathways through various mechanisms [42]. The lipid profile of patients with severe obesity shows a significant reduction in glycerophospholipids. Coupled with elevated levels of circulating fatty acids, this likely leads to impaired membrane remodelling. As a result, the biophysical properties of cells are affected, disrupting lipoprotein secretion and insulin sensitivity [21,43,44,45]. Given this context, the metabolic pathway of linolenic acid could be a promising therapeutic target. Animal studies have demonstrated that diet can modulate these lipid changes [46,47]. Although similar studies in humans are more complex, lipidomics and related immunosensing methods [48] may eventually serve as clinical tools for monitoring responses.

Weight loss following surgery results in a reduction in harmful lipid accumulation in plasma, confirming the pathogenic importance of dysfunctions in lipid fuel metabolism [19,49]. Specifically, fatty acids were significantly decreased, and glycerophospholipid species were restored, suggesting attenuated inflammation and improved membrane remodelling and lipid signalling. Similarly, lower levels of circulating sphingolipids and carnitines were revealed to enhance metabolic flexibility and reduce lipid storage. Our findings suggest that these lipid changes serve as biomarkers for metabolic recovery and reduced cardiovascular risk in patients [50,51,52]. A key finding of this study is that the circulating lipid profile does not completely revert to that of non-obese controls after a decrease in BMI. The lipidomic profiles can provide biomarkers to assess residual inflammation or adaptive immune remodelling, indicating an incomplete return to homeostasis [18,53,54]. Furthermore, the lower abundance of SMs in plasma is associated with a healthier composition of low-density lipoprotein and very low-density lipoprotein particles [55].

One strength of this study is the comparison of patients with differing outcomes. There is a need for further research into the mechanistic links between the benefits of weight loss and the significant changes in the circulating lipidome. In our patient population, we found a correlation between higher BMI and notable liver damage, as well as a poorer lipidomic response. This finding highlights the importance of prioritising liver health and implementing early interventions to prevent complications. The potential impact of gene variants is significant and deserves further investigation. The elevated levels of cholesteryl arachidonate in total responders indicate improved reverse cholesterol transport and enhanced the restoration of lipoprotein metabolism. Conversely, lower levels in partial responders could reflect persistent hepatic steatosis. Similarly, increased concentrations of glycerophospholipids such as PC 38:5, PC 36:4, and PE 38:5e in responders suggest active membrane remodelling, improved mitochondrial function, and enhanced lipid utilisation. In contrast, reduced levels in partial responders indicate impaired metabolic flexibility. The lower diacylglycerol (DG) concentrations observed in partial responders may reflect altered lipid storage or impaired lipolysis, potentially due to residual insulin resistance or deficient adipose tissue remodelling after surgery. Alternatively, higher DG levels in responders could be indicative of transient fat mobilisation. Overall, these lipidomic alterations point to disruptions in lipid transport mechanisms, possibly linked to changes in high-density and low-density lipoprotein composition, which may have implications for cardiovascular risk assessment [56].

The present study contributes to the growing body of literature on lipidomic alterations in obesity, offering novel and clinically relevant insights. The application of metabolomics to obesity is a relatively recent field. However, previous lipidomic studies have already demonstrated that obesity is associated with significant changes in circulating lipid species in humans and animal models [57,58]. For instance, elevated levels of ceramides, phosphatidylethanolamines, and phosphatidylinositols, alongside reduced concentrations of SMs, have been linked to metabolic dysfunction in children and adolescents with obesity [59]. Similar signatures have been reported in adults with severe obesity and type 2 diabetes mellitus [60,61]. Moreover, distinct lipid profiles have been proposed as potential markers of liver disease in individuals with severe obesity [62], and comparable alterations have been observed in human isolated adiposomes [63].

Our study builds upon and extends these findings by examining a well-characterised cohort of individuals with severe obesity, a population often presenting with pronounced metabolic derangements and multiple comorbidities. Unlike prior cross-sectional studies, our longitudinal design allowed us to track lipidomic changes one year after LSG, capturing the metabolic adaptations induced by substantial weight loss. Importantly, we found that patients who failed to reach a BMI below 35 kg/m^2^ after surgery (classified as partial responders) retained an unfavourable lipidomic profile. Although these individuals showed improvements in standard clinical chemistry parameters similar to those of total responders, their circulating lipid signatures remained distinct, suggesting persistent underlying metabolic alterations.

These findings highlight the potential of lipidomic profiling to identify individuals at risk of suboptimal metabolic response following bariatric surgery. While previous studies included patients with comparable degrees of obesity or comorbidities, our work adds to the literature by focusing on the dynamic metabolic response and stratifying patients according to their degree of postoperative improvement. This approach underscores the value of integrating lipidomic data into a broader framework of personalised risk assessment and therapeutic planning.

A natural question that arises is whether these findings can be translated into clinical practice. In our study, several lipid species were significantly associated with metabolic status both before and after LSG. Among those most strongly differentiating patients with obesity from lean controls were adrenic, palmitic, oleic, and palmitoleic acids. In contrast, cholesteryl arachidonate, specific glycerophospholipids, and diacylglycerols most effectively distinguished total from partial responders. Although these lipidomic signatures suggest potential for diagnosis or prognosis, their implementation in routine clinical set-tings remains technically challenging. Current diagnostic platforms do not routinely quantify individual lipid species at the resolution required for clinical decision-making. Nevertheless, our findings represent a step towards the future development of straight-forward and cost-effective lipid panels that may eventually support personalised follow-up strategies and risk stratification in patients undergoing bariatric surgery.

This study has several limitations. As it is an exploratory pilot study, our findings need to be validated in larger and more diverse cohorts. To enhance causal inference, we will apply Mendelian randomisation in future research designs. Additionally, to improve our methods, we will reassess the selection of lipid species to ensure that we do not overlook subtle but important changes in lipid profiles. Future studies should also consider longer-term metabolic adaptations and investigate the effects of other bariatric surgeries.

## 5. Conclusions

In summary, an integrative analysis of lipidomic and clinical markers shows that lipidomics can be a valuable tool for distinguishing between different metabolic outcomes and redefining success in weight loss. This discovery paves the way for the more precise treatment of severe obesity. Future studies will focus on translating the biomarker-guided strategy we propose into accessible diagnostic tools, ultimately improving patient outcomes beyond traditional metrics.

## Figures and Tables

**Table 1:** Characteristics of the study population ^1^.

Variable	Cohort 1 (n = 50)	Cohort 2 (n = 50)	Cohort 3 (n = 50)
Age, years	47 [36–58]	51 [43–57]	N/A
BMI, kg/m^2^	26.8 [23.0–28.9]	46.3 [43.4–53.8] ^a^	33.9 [31.2–37.5] ^a,b^
Waist circumference, cm	89.5 [79.2–103.2]	136.0 [127.0–145.0] ^a^	113.0 [104.5–121.6] ^b,c^
SBP, mmHg	125.0 [110.0–140.0]	138.0 [128.7–153.2] ^a^	132.5 [117.0–146.7]
DBP, mmHg	76.0 [70.0–85.0]	85.5 [73.7–94.0] ^a^	75.5 [67.0–87.0] ^c^
T2DM, n (%)	5 (10.0)	26 (52.0) ^a^	22 (44.0) ^b^
Hypertension, n (%)	7 (14.0)	50 (80.0) ^a^	33 (66.0) ^b^
Dyslipidemia, n (%)	3 (6.0)	26 (52.0) ^a^	26 (52.0) ^b^
Medication, n (%)			
Metformin	2 (4.0)	26 (52.0) ^a^	16 (32.0) ^b^
Sulfonylureas	1 (2.0)	7 (14.0)	6 (12.0)
Other T2DM	1 (2.0)	12 (24.0) ^a^	5 (10.0)
Insulin	1 (2.0)	11 (22.0) ^a^	6 (12.0)
ACEIs + ARA-II	2 (4.0)	26 (52.0) ^a^	25 (50.0) ^b^
Diuretics	1 (2.0)	12 (24.0) ^a^	13 (26.0) ^b^
Other AHT medications	0	15 (26.0) ^a^	14 (28.0) ^b^
Statins	1 (2.0)	17 (34.0) ^a^	15 (30.0) ^b^
Biochemical variables			
Glucose, mmol/L	4.8 [4.3–5.5]	7.9 [6.0–10.6] ^a^	4.7 [4.4–5.3] ^c^
Insulin, pmol/L	51.8 [32.7–67.5]	77.1 [55.6–145.1] ^a^	42.7 [27.8–67.7] ^c^
HOMA-IR	1.6 [1.0–2.4]	4.1 [2.4–7.9] ^a^	1.4 [0.9–2.0] ^c^
Triglycerides, mmol/L	1.2 [0.8–1.6]	1.8 [1.3–2.3] ^a^	1.0 [0.8–1.2] ^c^
Cholesterol, mmol/L	5.1 [4.5–6.0]	3.9 [3.3–4.7] ^a^	4.8 [4.4–5.6] ^c^
HDL, mmol/L	1.5 [1.2–1.7]	0.9 [0.7–1.0] ^a^	1.5 [1.3–1.8] ^c^
LDL, mmol/L	2.9 [2.5–3.9]	2.1 [1.7–2.8] ^a^	2.9 [2.5–3.2] ^c^
ALT, μKat/L	0.3 [0.2–0.4]	0.9 [0.5–1.2] ^a^	0.2 [0.2–0.3] ^b,c^
AST, μKat/L	0.3 [0.3–0.4]	0.8 [0.5–1.0] ^a^	0.3 [0.2–0.3] ^b,c^
GGT, μKat/L	0.2 [0.1–0.4]	0.5 [0.3–0.8] ^a^	0.2 [0.2–0.4] ^c^

^1^ Values are shown as number of cases and percentages or medians and interquartile ranges. ACEIs: Angiotensin-converting-enzyme inhibitors; AHT: Arterial hypertension; ALT: Alanine aminotransferase; ARA-II: Angiotensin II receptor antagonists; AST: Aspartate aminotransferase; BMI: Body mass index; DBP: Diastolic blood pressure; GGT: Gamma-glutamyl transferase; HDL: High-density lipoprotein; HOMA-IR: Homeostatic model assessment of insulin resistance; LDL: Low-density lipoprotein; SBP: Systolic blood pressure; T2DM: Type 2 diabetes mellitus. Significant differences (*p* ≤ 0.05 or lower) in comparisons are indicated by ^a^ Control vs. Severe Obesity. ^b^ Control vs. After Weight Loss and ^c^ Severe Obesity vs. After Weight Loss.

## Data Availability

The original contributions presented in this study are included in the article/Supplementary Materials. Further inquiries can be directed to the corresponding authors.

## Supplementary Materials

The following supporting information can be downloaded at: https://www.mdpi.com/article/10.3390/biom15081112/s1, Figure S1: Representative computed tomography images of study cohorts; Figure S2: Top discriminant lipid species across study group comparisons; Figure S3: Weight loss results in significant changes in lipid class composition; Figure S4: Lipidomic differences between patients with severe obesity after weight loss and the control group; Figure S5: Distribution of alleles among selected genotypes in partial and complete responders; Table S1: The 275 identified lipid species categorised by lipid family. Table S2: Detailed results from the circulating lipidomics analyses, comparing cohorts 1, 2, and 3, and including only those lipids with a fold change ≥ 1.5 and a *p*-value < 0.05. Table S3: Clinical, biochemical, and treatment characteristics of total and partial responders to laparoscopic sleeve gastrectomy; Table S4: Detailed results from the circulating lipidomics analyses, comparing complete and partial responders, and including only those lipids with a *p*-value < 0.05.

## Abbreviations

The following abbreviations are used in this manuscript:

BMI	Body mass index
DG	Diacylglycerol
FDR	False discovery rate
HETE	Hydroxyeicosatetraenoic acid
HOME	Hydroxyoctadecenoic acid
LPC	Lysophosphatidylcholine
LPE	Lysophosphatidylethanolamine
LSG	Laparoscopic sleeve gastrectomy
PC	Phosphatidylcholine
PLS-DA	Partial Least Squares Discriminant Analysis
QTOF	Quadrupole-time-on-flight mass spectrometry
ROC	Receiver Operating Characteristic
SM	Sphingomyelin
SNP	Single nucleotide polymorphism
UHPLC	Ultra-high-pressure liquid chromatography
VIP	Variable importance in projection
