# Correction: Onoiu et al. Circulating Lipid Profiles Indicate Incomplete Metabolic Recovery After Weight Loss, Suggesting the Need for Additional Interventions in Severe Obesity. *Biomolecules* 2025, *15*, 1112

**DOI:** 10.3390/biom16010034

**Published:** 2025-12-25

**Authors:** Alina-Iuliana Onoiu, Vicente Cambra-Cortés, Andrea Jiménez-Franco, Anna Hernández-Aguilera, David Parada, Francesc Riu, Antonio Zorzano, Jordi Camps, Jorge Joven

**Affiliations:** 1 Unitat de Recerca Biomèdica, Hospital Universitari de Sant Joan, Institut d’Investigació Sanitària Pere Virgili, Universitat Rovira i Virgili, Av. Dr. Josep Laporte 2, 43204 Reus, Spain; alinaiuliana.onoiu@urv.cat (A.-I.O.); vicente.cambra@urv.cat (V.C.-C.); andrea.jimenez@urv.cat (A.J.-F.); 2 Department of Pathology, Hospital Universitari de Sant Joan, Institut d’Investigació Sanitària Pere Virgili, Universitat Rovira i Virgili, Av. Dr. Josep Laporte 2, 43204 Reus, Spain; anna.hernandez@salutsantjoan.cat (A.H.-A.); david.parada@salutsantjoan.cat (D.P.); francesc.riu@salutsantjoan.cat (F.R.); 3 Institute for Research in Biomedicine, Department of Biochemistry and Molecular Medicine, Universitat de Barcelona, C. Baldiri Reixac 10, 08028 Barcelona, Spain; antonio.zorzano@irbbarcelona.org

In the original publication [1], there were mistakes in Figures 1C and 4C as published. Figure 1C incorrectly displays a mirrored volcano plot, and there was a mistake regarding the order of Figures 1C and 4C. The corrections regarding Figure 1C and Figure 4C are provided below.

The authors state that the scientific conclusions are unaffected. This correction was approved by the Academic Editor. The original publication has also been updated.

## Figures and Tables

**Figure 1:**
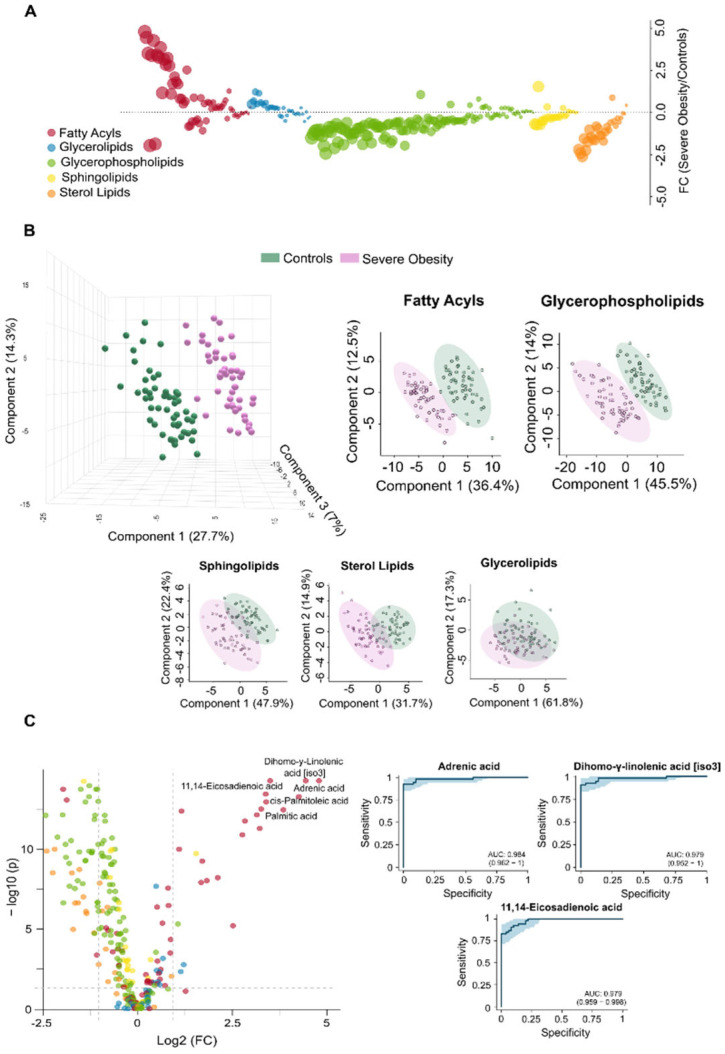
Severe obesity induces significant changes in the circulating lipidome. (**A**) The bubble plot illustrates the magnitude and direction of lipid alterations across different classes in individuals with severe obesity compared to controls. (**B**) The Partial Least Squares Discriminant Analysis shows a clear distinction between the control group and individuals with severe obesity across various lipid classes, with fatty acyls and glycerophospholipids demonstrating the strongest ability to differentiate between the groups. (**C**) The volcano plot displays the individual lipids showing the greatest differences between groups, and the Receiver Operating Characteristic curves for the three most significantly altered species, highlight their potential as diagnostic indicators of metabolic stress. AUC: Area under the curve; FC: Fold change.

**Figure 4:**
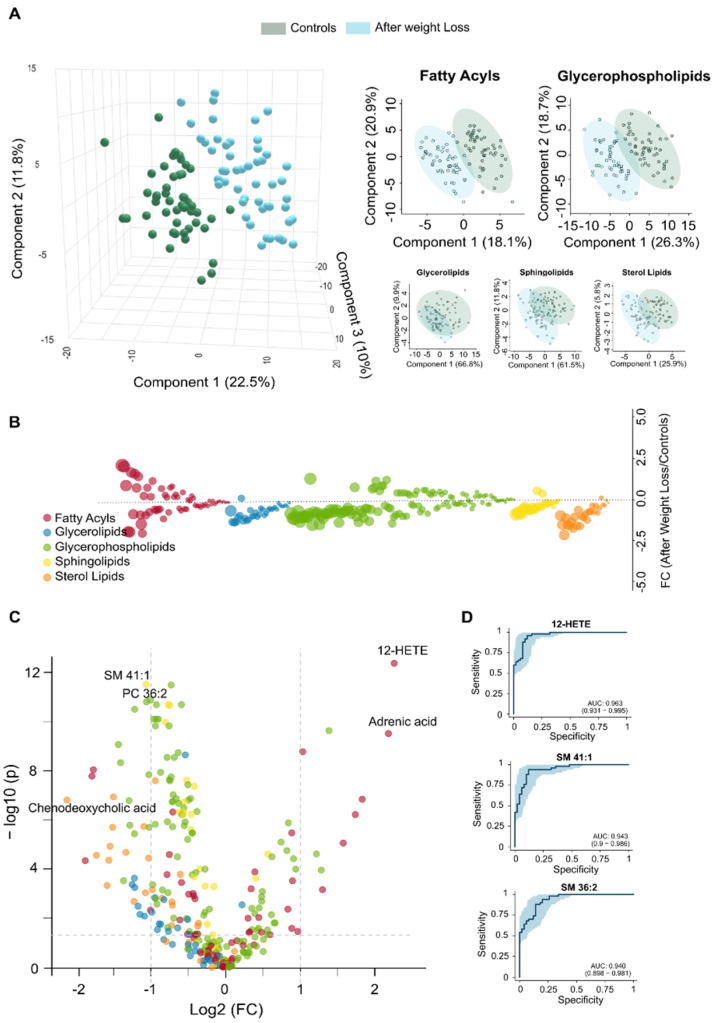
Comparisons in the circulating lipidome between post-surgical patients and control group confirm metabolic recovery. (**A**) The two groups show a modest separation in the Partial Least Squares Discriminant Analysis, with no lipid family exhibiting complete separation. (**B**) The bubble plot displays the magnitude and direction of remaining lipid alterations across families. (**C**) The volcano plot illustrates the differentially abundant lipids species between the two groups. (**D**) Receiver Operating Characteristic analysis highlights the top three discriminating lipid biomarkers, revealing residual alterations in lipid homeostasis. AUC: Area under the curve; FC: Fold change; HETE: Hydroxyeicosatetraenoic acid; SM: Sphingomyelin.
