# Supplementary material for: Circulating Lipid Profiles Indicate Incomplete Metabolic Recovery After Weight Loss, Suggesting the Need for Additional Interventions in Severe Obesity
Source: Biomolecules. 2025 Aug 1;15(8):1112. doi: 10.3390/biom15081112 (PMC12383904; doi:10.3390/biom15081112)
Supplement: Supplementary file 1 [file biomolecules-15-01112-s001.zip › Supplementary Table S3.pdf]

Supplementary Table S3. Clinical, biochemical, and treatment characteristics of total and partial responders to laparoscopic sleeve gastrectomy.

Variable	Total responders (n = 29)	Partial responders (n = 21)	p-value
Age, years	53 [42-65]	47 [42-57]	0·151
Baseline BMI, kg/m^2	46·4 [42·9-53·1]	55·4 [50·0-57·3]	0·001
Post-Surgery BMI, kg/m^2	31·4 [30·2-33·2]	38·4 [37·1-41·9]	2·97x10^{-14}
Waist circumference, cm	106·0 [100·0-110·8]	120·2 [116·2-126·8]	2·48x10^{-5}
T2DM, n (%)	14 (48·3)	8 (38·0)	0·669
Hypertension, n (%)	18 (62·1)	15 (71·4)	0·699
Dyslipidaemia, n (%)	16 (55·2)	10 (47·6)	0·810
Medication, n (%)			
Metformin	10 (34·5)	6 (28·6)	0·892
Sulfonylureas	5 (17·2)	1 (4·8)	0·368
Other T2DM	3 (10·3)	2 (9·5)	1
Insulin	5 (17·2)	1 (4·8)	0·368
ACEIs+ARA II	13 (44·8)	12 (57·1)	0·567
Diuretics	10 (34·5)	3 (14·3)	0·200
Other AHT medications	11 (37·9)	3 (14·0)	0·129
Statins	12 (41·4)	3 (14·0)	0·080
Biochemical variables			
Glucose, mmol/L	4·6 [4·3-5·0]	4·8 [4·5-6·5]	0·602
Insulin, pmol/L	39·6 [27·8-59·7]	50·0 [35·4-68·1]	0·262
HOMA-IR	1·3 [0·7-2·0]	1·4 [1·1-2·0]	0·582
Triglycerides, mmol/L	0·9 [0·8-1·2]	1·0 [0·9-1·2]	0·504
Cholesterol, mmol/L	4·8 [4·4-5·7]	4·8 [4·5-5·2]	0·761
HDL, mmol/L	1·5 [1·3-1·8]	1·6 [1·2-1·6]	0·816
LDL, mmol/L	2·9 [2·6-3·3]	2·8 [2·4-3·2]	0·537
ALT, μKat/L	0·2 [0·2-0·3]	0·2 [0·2-0·4]	0·443
AST, μKat/L	0·3 [0·2-0·3]	0·3 [0·3-0·4]	0·616
GGT, μKat/L	0·2 [0·1-0·3]	0·3 [0·2-0·4]	0·198

Values are shown as number of cases and percentages or medians and interquartile ranges. ACEIs: Angiotensin-converting-enzyme inhibitors; AHT: Hypertension; ALT: Alanine aminotransferase; ARA-II: Angiotensin II receptor antagonists; AST: Aspartate aminotransferase; BMI: Body mass index; DBP: Diastolic blood pressure; GGT: Gamma-glutamyl transferase; HDL: High-density lipoprotein; HOMA-IR: Homeostatic model assessment of insulin resistance; LDL: Low-density lipoprotein; SBP: Systolic blood pressure; T2DM: Type 2 diabetes mellitus.
