# Supplementary material for: Circulating Lipid Profiles Indicate Incomplete Metabolic Recovery After Weight Loss, Suggesting the Need for Additional Interventions in Severe Obesity
Source: Biomolecules. 2025 Aug 1;15(8):1112. doi: 10.3390/biom15081112 (PMC12383904; doi:10.3390/biom15081112)
Supplement: Supplementary file 1 [file biomolecules-15-01112-s001.zip › Supplementary_Figures.pdf]

Supplementary figures corresponding to the article:

The circulating lipid profiles indicate incomplete metabolic recovery after weight loss, suggesting the need for additional interventions in severe obesity

Alina-Iuliana Onoiu,[a,b] Vicente Cambra-Cortés,[a] Andrea Jiménez-Franco,[a,b] Anna Hernández-Aguilera,[c] David Parada,[c] Francesc Riu,[c] Antonio Zorzano,[d] Jordi Camps,[a,b,]* and Jorge Joven,[a,b,e,]*

[a] *Unitat de Recerca Biomèdica, Hospital Universitari de Sant Joan, Institut d'Investigació Sanitària Pere Virgili, Universitat Rovira i Virgili, 43204 Reus, Spain.*
[b] *School of Medicine, Universitat Rovira i Virgili, 43201 Reus, Spain.*
[c] *Department of Pathology, Hospital Universitari de Sant Joan, Institut d'Investigació Sanitària Pere Virgili, Universitat Rovira i Virgili, 43204 Reus, Spain.*
[d] *Department of Biochemistry and Molecular Medicine, Universitat de Barcelona, Barcelona, Spain*
[e] *The Campus of International Excellence Southern Catalonia, 43003 Tarragona, Spain.*

* Corresponding authors. Unitat de Recerca Biomèdica, Hospital Universitari de Sant Joan, Av. Dr. Josep Laporte 2, 43204 Reus, Spain
E-mail addresses: jorge.camps@salutsantjoan.cat (J. Camps);jorge.joven@salutsantjoan.cat (J.Joven).

Index:

**Figure**	**Page**
Supplementary Figure 1	2
Supplementary Figure 2	3
Supplementary Figure 3	4
Supplementary Figure 4	5
Supplementary Figure 5	6

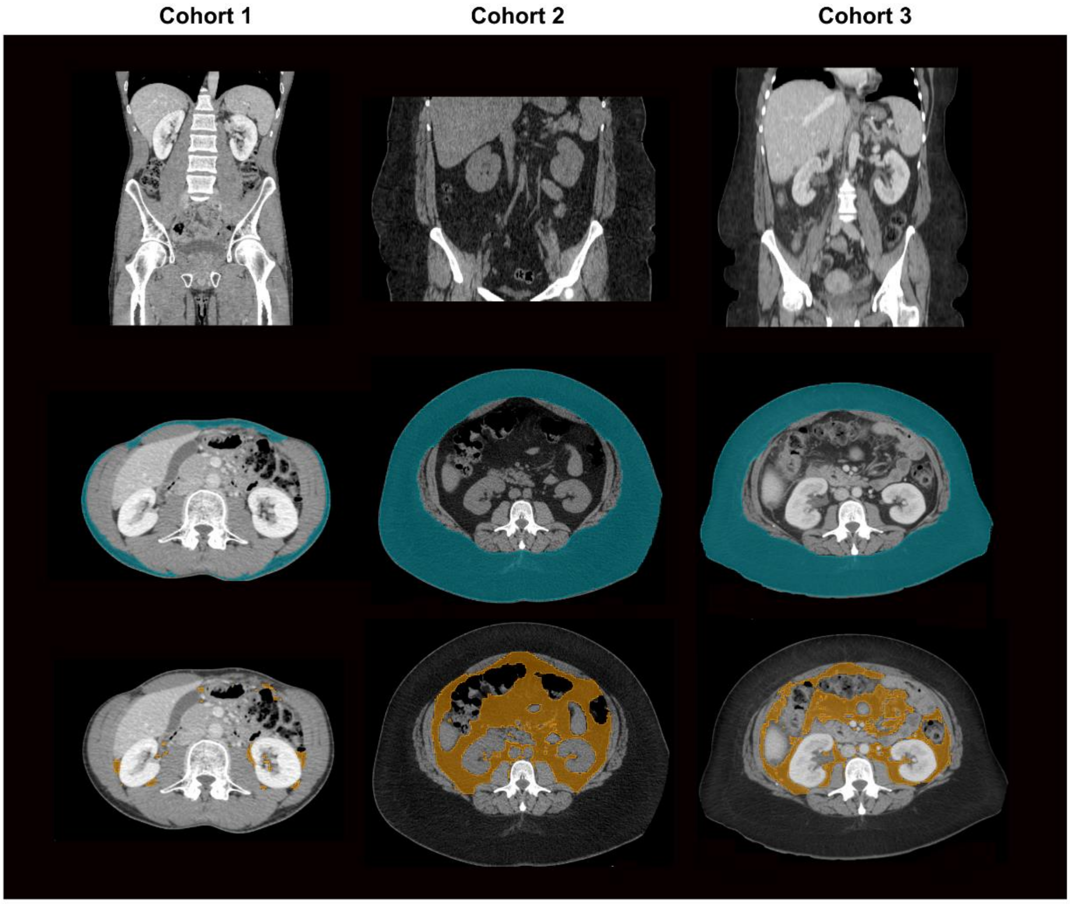

Supplementary Fig. S1. Representative computed tomography images of study cohorts.
This figure presents illustrative coronal and axial computed tomography slices for the three study groups: Cohort 1 (Control Group), Cohort 2 (Severe Obesity Group), and Cohort 3 (Post-Surgery Group). The second and third rows feature axial slices with overlays that highlight subcutaneous adipose tissue and visceral adipose tissue. These images clearly show the differences in adipose tissue distribution and volume among the cohorts, particularly the significant reduction in visceral fat observed in Cohort 3.

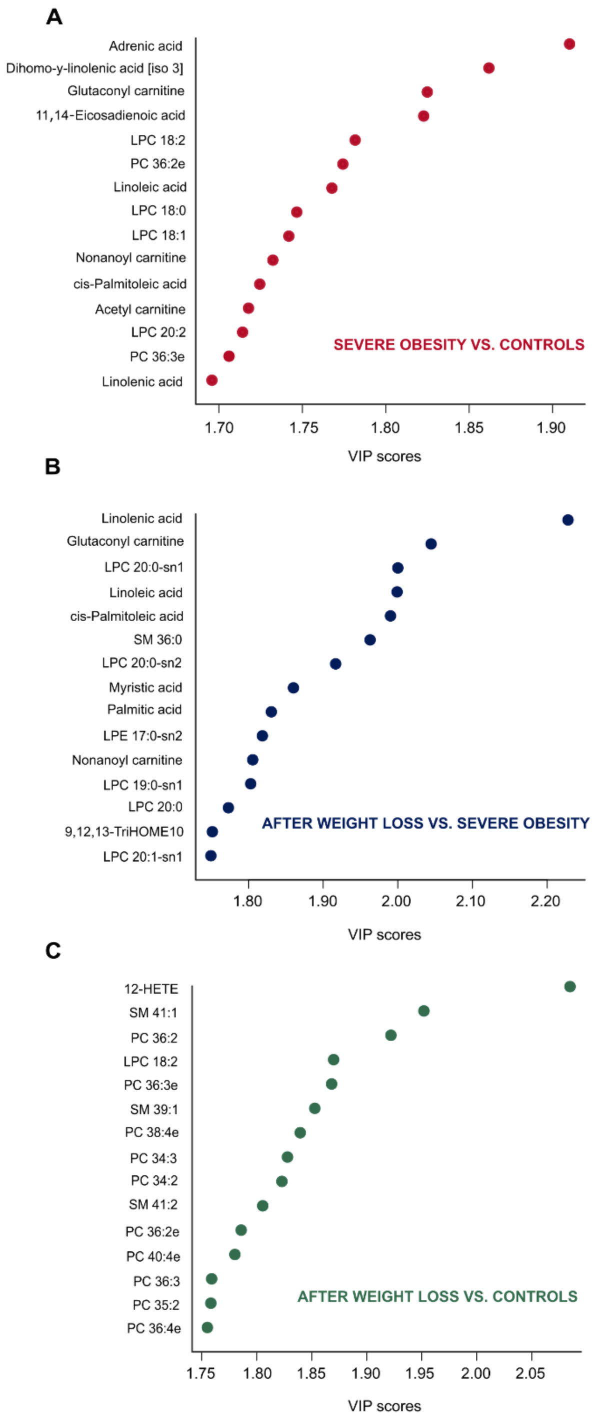

Supplementary Fig. S2. Top discriminant lipid species across study group comparisons. Each plot displays lipid species with the highest variable importance in the projection (VIP) scores, indicating their importance in differentiating between groups: (A) Severe obesity vs. controls (red), (B) After weight loss vs. severe obesity (blue), and (C) After weight loss vs. controls (green).
HETE: Hydroxyeicosatetraenoic acid; LPC: Lysophosphatidylcholine; PC: Phosphatidylcholine; SM: Sphingomyelin; TriHOME: Trihydroxyoctadecenoic acid.

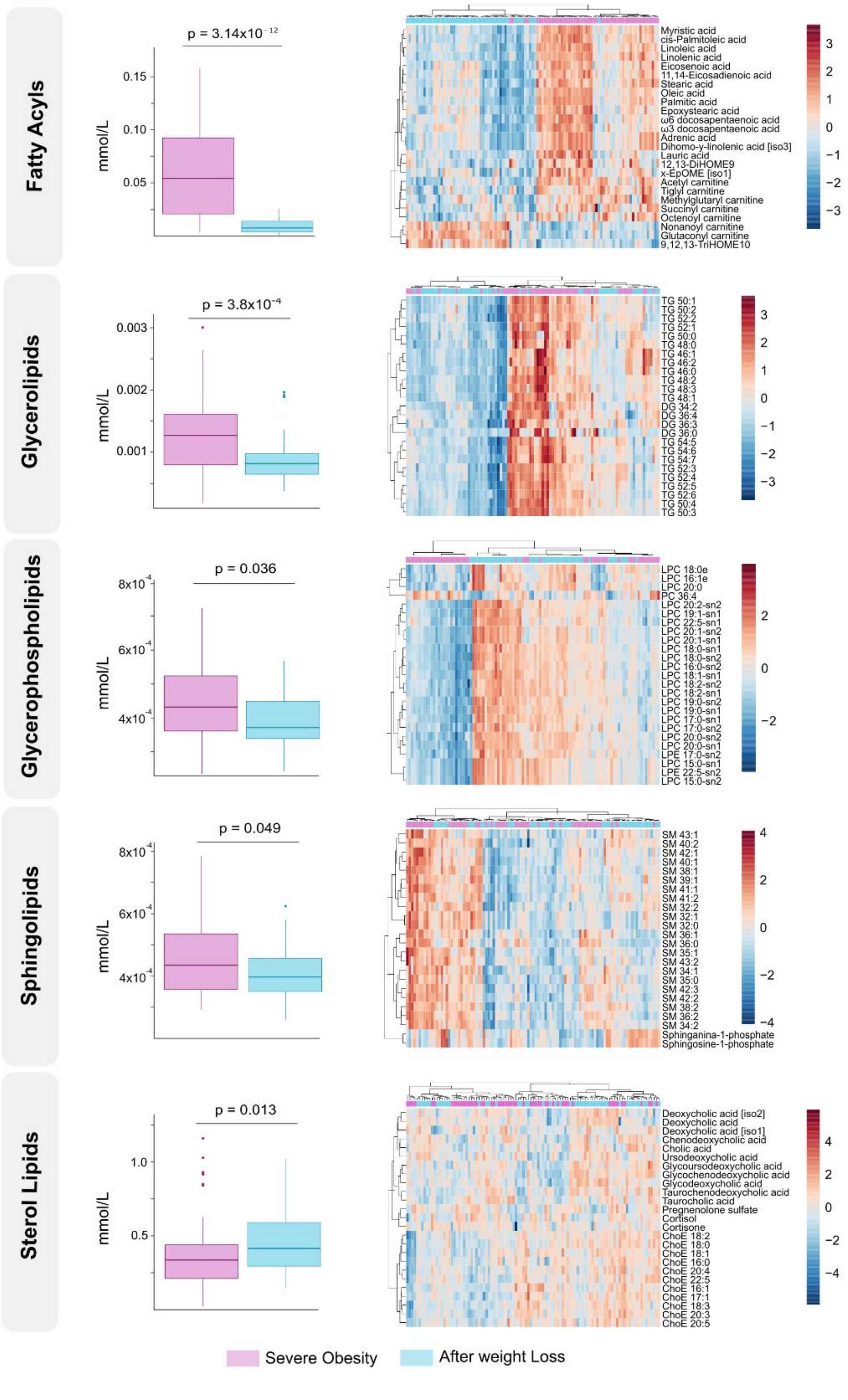

Supplementary Fig. S3. Weight loss results in significant changes in lipid class composition. The box plots on the left display lipid classes in patients with severe obesity before (pink) and after weight loss (blue). On the right, heatmaps show the 25 most differentially expressed lipid species within each class. Hierarchical clustering reveals distinct regulatory patterns associated with weight loss. ChoE: Cholesterol ester; DG: Diglyceride; DiHOME: Dihydroxyoctadecenoic acid; EpOME: Epoxide form of linoleic acid; LPC: Lysophosphatidylcholine; PC: Phosphatidylcholine; SM: Sphingomyelin; TG: Triglyceride; TriHOME: Trihydroxyoctadecenoic acid.

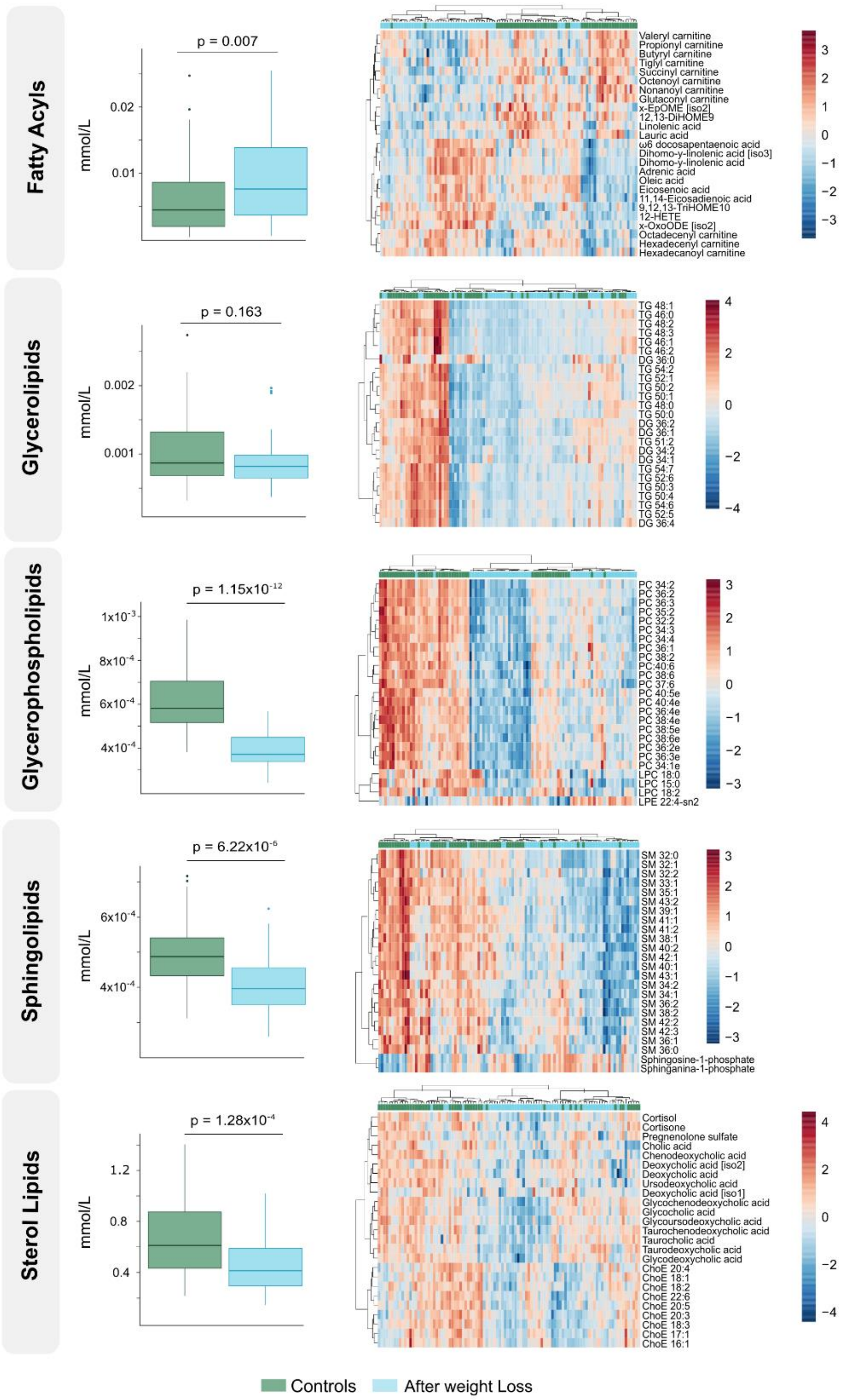

Supplementary Fig. S4. Lipidomic differences between patients with severe obesity after weight loss and the control group.

The box plots on the left display the concentrations of major lipid classes in the control group (green) and in patients with severe obesity after weight loss (blue). On the right, the heatmaps illustrate the 25 most differentially expressed lipid species within each class, with hierarchical clustering highlighting distinct patterns of lipid regulation between the two groups.

ChoE: Cholesterol ester; DG: Diglyceride; DiHOME: Dihydroxyoctadecenoic acid; EpOME: Epoxide form of linoleic acid; LPC: Lysophosphatidylcholine; LPE: Lysophosphatidylethanolamine; oxoODE: oxooctadecadienoic acid; PC: Phosphatidylcholine; SM: Sphingomyelin; TG: Triglyceride; TriHOME: Trihydroxyoctadecenoic acid.

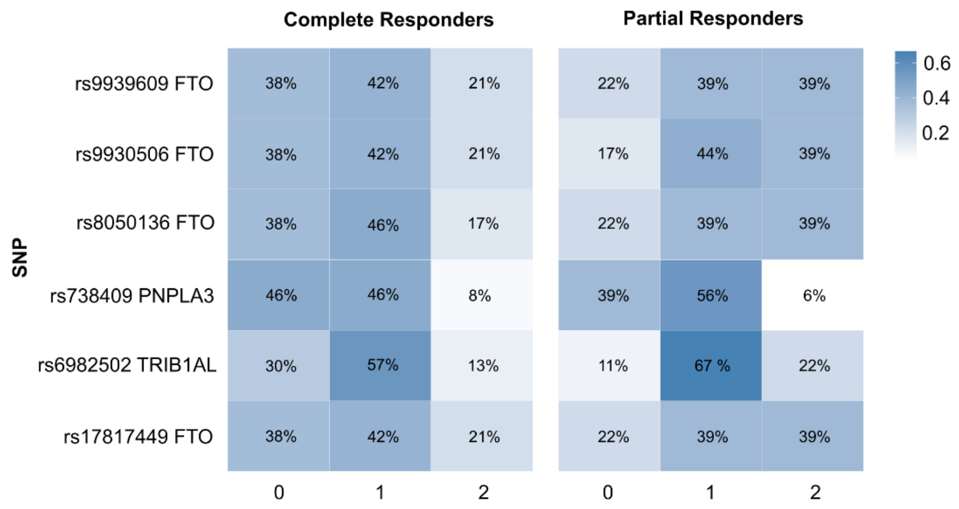

Supplementary Fig. S5. Distribution of alleles among selected genotypes in partial and complete responders.

This figure illustrates the distribution of genotypes across selected loci within groups of partial and complete responders. Genotypes are categorised as follows: 0 for homozygous for the common allele, 1 for heterozygous, and 2 for homozygous for the less common, pathogenic allele. The percentages within each genotype group highlight the differences in the frequency of genetic variants.

FTO: Fat mass and obesity-associated gene; *PNPLA3*: Patatin-like phospholipase domain-containing protein 3; *TRIB1AL*: Tribbles pseudokinase 1-associated lnc RNA.
